# Biofilm formation of methicillin-resistant coagulase negative staphylococci (MR-CoNS) isolated from community and hospital environments

**DOI:** 10.1371/journal.pone.0184172

**Published:** 2017-08-31

**Authors:** Rathanin Seng, Thawatchai Kitti, Rapee Thummeepak, Phattaraporn Kongthai, Udomluk Leungtongkam, Surat Wannalerdsakun, Sutthirat Sitthisak

**Affiliations:** 1 Department of Microbiology and Parasitology, Faculty of Medical Science, Naresuan University, Phitsanulok, Thailand; 2 Faculty of Oriental Medicine, Chiang Rai College, Chiang Rai, Thailand; 3 Department of Medicine, Division of Infectious Diseases, Faculty of Medicine, Naresuan University, Phitsanulok, Thailand; 4 Centre of Excellence in Medical Biotechnology, Faculty of Medical Science, Naresuan University, Phitsanulok, Thailand; Rockefeller University, UNITED STATES

## Abstract

Methicillin-resistant coagulase negative staphylococci (MR-CoNS) are the major cause of infectious diseases because of their potential ability to form biofilm and colonize the community or hospital environments. This study was designed to investigate the biofilm producing ability, and the presence of *mecA*, *ica*AD, *bap* and *fnb*A genes in MR-CoNS isolates. The MR-CoNS used in this study were isolated from various samples of community environment and five wards of hospital environments, using mannitol salt agar (MSA) supplemented with 4 μg/ml of oxacillin. The specie level of *Staphylococcus haemolyticus*, *Staphylococcus epidermidis*, *Staphylococcus hominis* and *Staphylococcus warneri* was identified by specific primers of *groESL* (*S*. *haemolyticus*), *rdr* (*S*. *epidermidis*) and *nuc* (*S*. *hominis* and *S*. *warneri*). The remainder isolates were identified by *tuf* gene sequencing. Biofilm production was determined using Congo red agar (CRA) and Microtiter plate (MTP) assay. The *mecA* and biofilm associated genes (*ica*AD, *fnb*A and *bap*) were detected using PCR method. From the 558 samples from community and hospital environments, 292 MR-CoNS were isolated (41 from community environments, and 251 from hospital environments). *S*. *haemolyticus* (41.1%) and *S*. *epidermidis* (30.1%) were the predominant species in this study. Biofilm production was detected in 265 (90.7%) isolates by CRA, and 260 (88.6%) isolates were detected by MTP assay. The staphylococci isolates derived from hospital environments were more associated with biofilm production than the community-derived isolates. Overall, the *ica*AD and *bap* genes were detected in 74 (29.5%) and 14 (5.6%) of all isolates from hospital environments. When tested by MTP, the *ica*AD gene from hospital environment isolates was associated with biofilm biomass. No association was found between *bap* gene and biofilm formation. The MR-CoNS isolates obtained from community environments did not harbor the *ica*AD and *bap* genes. Conversely, *fnb*A gene presented in MR-CoNS isolated from both community and hospital environments. The high prevalence of biofilm producing MR-CoNS strains demonstrated in this study indicates the persisting ability in environments, and is useful in developing prevention strategies countering the spread of MR-CoNS.

## Introduction

Coagulase-negative staphylococci (CoNS) are opportunistic pathogens that persist and multiply on a variety of environmental surfaces. It is the cause of both nosocomial and community acquired infections worldwide [1]. Additionally, CoNS develops resistance to various antimicrobial agents causing difficulties in treatment strategies [2]. The prevalence of methicillin-resistant coagulase negative staphylococci (MR-CoNS) has been reported [3]. In addition, biofilm production by CoNS has been identified as an important factor of pathogenesis, protecting against antibiotics and the immune system [4]. The biofilm consists of layers of cell clusters embedded in a matrix of extracellular polysaccharide, called polysaccharide intracellular adhesion (PIA) [5]. The development of the biofilm is considered to be a two-step process; beginning with bacteria adhering to a biotic or an abiotic surface mediated by microbial surface components recognizing adhesive matrix molecules (MSCRAMMs) [6]. Then, the bacteria multiply to form a multilayered biofilm, associated with production of PIA which mediates cell to cell adhesion [7]. The synthesis of PIA is mediated by the products of the intracellular adhesion (*ica*) operon. This operon contains *ica*ABCD genes involved in the synthesis of a biofilm matrix polysaccharide (named PIA/PNAG), composed of linear β-1-6-linked *N*-acetyl glucosamine residues [8]. Biofilm-associated protein (Bap) has been shown to be involved in the initial attachment, intracellular adhesion, and biofilm formation. It has been reported that Bap-positive isolates become resistant to antibiotic treatments when forming biofilms [9]. Bap induces an alternative mechanism of biofilm formation also found in *S*. *epidermidis* [10]. The fibronectin binding proteins (FnbA) play an important role in the accumulation phase of biofilm formation. FnbA promotes biofilms through homophilic interactions or through binding of the proteins to surface-located receptors on adjacent cells [11]. Previous studies have reported the prevalence of CoNS isolates recovered from hospital environments (air, walls, floors and medical equipment); frequently found were *S*. *epidermidis* (26.2%), *S*. *haemolyticus* (25.4%), *S*. *capitis* (17.2%) and 20 .5% of CoNS isolates were able to produce biofilm by Microtiter Plate method [12]. In addition, biofilm production was observed in *S*. *epidermidis* in 8.7% (8/92) of the nasal isolates from healthcare staff (doctors and nurses), and 6.3% (7/112) from healthy volunteers in the Shanghai area of China [13].

However, the prevalence of biofilm formation of MR-CoNS isolated from environments is rarely reported in Thailand. It is necessary to identify the genetic determinants of virulence which are important in biofilm formation. In this study, we investigated the biofilm production and the presence of adhesin genes *icaAD*, *bap* and *fnb*A in the MR-CoNS isolated from community and hospital environments in Naresuan University, Phitsanulok province, Thailand.

## Materials and methods

### Sample

MR-CoNS were isolated from 358 samples from hospital environments, and 200 from community environments. The hospital environment samples were collected from the surfaces of medical trolleys (n = 50), intravenous poles (n = 50), patient beds (n = 50), wash-basins (n = 50), door handles (n = 40), stethoscopes (n = 50), and nurse stations (n = 50) from five departments of a university hospital. All five departments were the outpatient department, the emergency room, the medicine ward, the surgical ward, and the intensive care unit. Additionally, eighteen swab samples were collected from laboratory clothes of medical students. Samples from surfaces that are frequently touched by people were randomly collected from 5 regions of a university community in Naresuan University, Thailand. The source of the samples included those from computer rooms (computer mouses, computer earpieces, computer keyboards and computer power buttons) (N = 40), restrooms (door handles, wash-basins, wash-basin areas, urinal taps and toilets) (N = 50), the library (books, escalators and tables) (N = 30), canteens (tables, bank notes and coins used for payment, ATM machines and water dispensers) (N = 40), and outdoor surfaces (handrails, exercise machines, public buses) (N = 40).

### Isolation and identification of methicillin-resistant coagulase negative staphylococci (MR-CoNS) from community and hospital environments

The environmental surfaces were collected using cotton swab soaked in 0.85% normal saline, then placed in the transfer media (2% of skim milk powder, 3% of tryptone soya broth, 0.5% Glucose and 10% Glycerol). The swab samples were enriched in Tryptone Soya broth with shaking at 180 rpm and 37 ^o^C for 18–24 hours, and then cultured in Mannitol Salt Agar with 4 mg/ml of oxacillin at 30 ^o^C for 48–72 hours. Cultures with yellow and white colonies were selected for further evaluation using Gram’s stain, catalase and coagulase tests (BD Diagnostics, USA). All isolates were subsequently confirmed as staphylococci by polymerase chain reaction (PCR) using 16S rRNA primers specific to staphylococci [14]. The isolates were stored in Tryptone Soya broth, to which 20% sterile glycerol was added, at -20 ^o^C.

### Species identification of methicillin-resistant coagulase negative staphylococci

*S*. *epidermidis*, *S*. *haemolyticus*, *S*. *hominis and S*. *warneri* were distinguished from other staphylococcal species by PCR based method on the specific primers of *groESL* (*S*. *haemolyticus*), *rdr* (*S*. *epidermidis*) and *nuc* (*S*. *hominis and S*. *warneri*), as described by Schmidt, Kock and Ehlers (2015) [15]. A specific gene of each species was sequenced to ensure the absence of bias in our method. The primer sets of *rdr*, *groESL* and *nuc* genes are shown in Table 1. The isolates that could not be amplified by PCR were further identified using *tuf* gene sequencing [15]. Methicillin-resistance was then confirmed by oxacillin disk (1 μg), cefoxitin disk (30 μg), and PCR to detect *mec*A gene as described by Kitti et al (2011) [16]. PCR assay was performed in a DNA thermal cycler (GeneMate). The amplified PCR products were analyzed on a 1% agarose gel.

**Table 1:** List of primers used in this study.

Target gene	Forward primer	Reverse primer	Size (bp)	Tm (^o^C)	Reference
*16S* rRNA	CGAAAGCCTGACGGAGCAAC	AACCTTGCGGTCGTACTCCC	528	52	[14]
*tuf*	CCAATGCCACAAACTCGTGA	CAGCTTCAGCGTAGTCTAATAATTTACG	480	62	[15]
*rdr* (*S*. *epidermidis*)	AAGAGCGTGGAGAAAAGTATCAAG	TCGATACCATCAAAAAGTTGG	130	61.8	[38]
*groESL* **(***S*. *haemolyticus***)**	GGTCGCTTAGTCGGAACAAT	CACGAGCAATCTCATCACCT	271	57.8	[39]
*nuc* (*S*. *hominis*)	TACAGGGCCATTTAAAGACG	GTTTCTGGTGTATCAACACC	177	56.4	[40]
*nuc (S*. *warneri)*	CGTTTGTAGCAAAACAGGGC	GCAACGAGTAACCTTGCCAC	999	59	[40]
*mec*A	TGGCTATCGTGTCACAATCG	CTGGAACTTGTTGAGCAGAG	310	58	[41]
*ica*AD	GACAGTCGCTACGAAAAG	AATAAGCTCTCCCTAACTA	211	55	This study
*fnb*A	CCCTCTTCGTTATTCAGCC	CAGGAGGCAAGTCACCTTG	422	58	This study
*bap*	GGCGCAAGCAGCAGAATTA	CATAGTTCTTTGTGGTGTTGC	901	63	This study

### Determination of antibiotic susceptibility

The antibiotic susceptibility patterns of penicillin (P, 10 units), clindamycin (DA; 2 μg), chloramphenicol (C; 30 μg), gentamicin (CN; 10 μg), erythromycin (E; 15 μg), cefoxitin (FOX; 30 μg), sulfamethoxazole/trimethoprim (SXT; 1.25/23.75 μg), vancomycin (VA; 30 μg), rifampicin (RD; 5 μg), linezolid (LZD; 30 μg), mupirocin (MUP; 5 μg), ciprofloxacin (CIP; 5 μg), fusidic acid (FD; 10 μg) and novobiocin (NV; 5 μg) (Oxoid) were determined according to the antibiotic disk diffusion method [17]. The plates were incubated at 35°C for 24 hours. The zones of inhibition were determined whether the microorganism was susceptible, intermediately resistant, or resistant to each antibiotic.

### Study of biofilm production

#### Detection of biofilm production of MR-CoNS by the Congo red agar (CRA) method

Biofilm production was performed on Congo red agar (CRA) plates as described by Freeman and coworkers [18]. The isolates were streaked on the CRA plate and incubated at 35°C under aerobic conditions for 24 to 48 hours. The staphylococci biofilm producer strains formed black and very black colonies and the non-biofilm producer strains formed red colonies. Black was indicated by a darkening of colony with the absent of dry crystalline colonial morphology, while very black isolates formed a darkening of colony with the present of dry crystalline.

#### Detection of biofilm production of MR-CoNS by the microtiter plate (MTP) method

Quantitative microtiter plate (MTP) assay for biofilm formation was performed as described by Bekir et al (2011) [19]. MR-CoNS isolates were cultivated overnight in 96-well polystyrene tissue culture microtiter plates (Nunc, Denmark) at 37°C, with trypticase soy broth supplemented with 0.25% glucose as the growth medium. After incubation, the culture medium was removed and adherent cells were fixed with 95% ethanol, and stained with 1% crystal violet. Absorbance at 570 nm was determined. Isolates are considered biofilm-positive when they have an OD_570 nm_ > 0.1. Each isolate was tested in triplicate. Biofilm formation was interpreted as follows: highly positive (OD570 ≥ 1), low grade positive (0.1 ≤ OD570 < 1), or negative (OD570 < 0.1) (Tangchaisuriya *et al*., 2014).

#### Molecular detection of biofilm associated genes

The DNA sequences of the *ica*AD (accession no. SEU43366, FJ472951, KJ544506 and AF246926), *fnb*A (accession no. Y17116, AF245042 and AF245042) and *bap* (accession no. DQ008306, EU011246 and HQ170520) were taken from the GenBank Sequence Database of NCBI (http://www.ncbi.nlm.nih.gov) and used as a template to design primers (listed in Table 1). Primers specific to the conserved region of each virulence gene used in this study were manually designed by using primer-BLAST software (https://www.ncbi.nlm.nih.gov/tools/primer-blast/). The boiled cell lysates were used as a DNA template for PCR amplification of each gene. PCR assay was performed in a DNA thermal cycler (GeneMate). The amplified PCR products were analyzed on a 1% agarose gel.

### Statistical analysis

Statistical data and comparisons were analyzed by using Stata (Stata12.0, Corporation,USA). The median OD_570_ was calculated and expressed with interquartile range (IQR). The comparison of median values between two groups was performed by Mann–Whitney U test. In case of non-parametric tests of three or more groups, Kruskal-Wallis and Dunn's test were performed to compare the median values among multiple groups.

## Results

### Isolation of methicillin-resistant coagulase negative staphylococci

A total of 292 MR-CoNS isolates from community and hospital environments were isolated in this study. The prevalence of MR-CoNS isolated from community environments was 20.5% (41/200). The library was the most contaminated, with 43.3% of the regions tested showing staphylococci contamination. We found 70.1% (251/358) of environmental regions in hospital was colonized by MR-CoNS, in which laboratory clothes were the most contaminated, with 94.4% of the tested samples showing MR-CoNS contamination. The next most contaminated regions were the medicine ward (84.3%), the intensive care unit (75.7%), the emergency room (74.3%), the surgical ward (71.4%), and the outpatient department (33.3%), respectively. All 292 isolates obtained from the community and hospital environments belonged to *S*. *haemolyticus* (41.1%), *S*. *epidermidis* (30.1%), *S*. *capitis* (11.3%), *S*. *warneri* (9.6%), *S*. *cohnii* (2.7%), *S*. *pasteuri* (1.0%), *S*. *caprae* (0.7%), *S*. *hominis* (0.7%), *S*. *saprophyticus* (0.7%), *S*. *nepalensis* (0.3%) and unidentified *Staphylococcus* spp. (1.7%) (S1 Table**).**

### Antibiotic susceptibility testing

All MR-CoNS isolates were tested for antimicrobial sensitivities against 14 antibiotics and all of them showed the resistance to at least one antibiotic class. The isolates were resistant to penicillin (99.3%), cefoxitin (94.9%), erythromycin (82.9%), clindamycin (67.1%), sulfamethoxazole/trimethoprim (43.5%), ciprofloxacin (41.4%), gentamicin (36.3%), fusidic acid (19.5%), rifampicin (12.0%), mupirocin (8.6%), chloramphenicol (6.8%), novobiocin (2.4%), and linezolid (0.3%). However, all isolates were sensitive to vancomycin **(**Table 2). The *mec*A gene was detected in all MR-CoNS isolates (Table 3).

**Table 2:** Drug resistance of MR-CoNS isolated from community and hospital environments.

Antibiotics	Community environment			Hospital environment			Total (%) n = 292
	SE (%) n = 15	SH (%) n = 17	OT (%) n = 9	SE (%) n = 73	SH (%) n = 103	OT (%) n = 75	
Penicillin	15(100)	17(100)	9(100)	72(98.6)	103(100)	74(98.7)	290(99.3)
Cefoxitin	7(46.7)	15(88.2)	8(88.9)	71(97.3)	101(98.0)	75(100)	277(94.9)
Erythromycin	11(73.3)	11(64.7)	8(88.9)	59(80.8)	89(86.4)	64(85.3)	242(82.9)
Sulfamethoxazole/Trimethoprim	5(33.3)	3(17.6)	1(11.1)	36(49.3)	60(58.2)	22(29.3)	127(43.5)
Fusidic acid	2(13.3)	1(5.9)	3(33.3)	18(24.7)	14(13.6)	19(25.3)	57(19.5)
Clindamycin	7(46.7)	2(11.8)	3(33.3)	53(72.6)	76(73.8)	55(73.3)	196(67.1)
Ciprofloxacin	2(13.3)	2(11.8)	1(11.1)	37(50.7)	56(54.4)	23(30.7)	121(41.4)
Chloramphenicol	1(6.7)	2(11.8)	1(11.1)	6(8.2)	4(3.9)	6(8.0)	20(6.8)
Novobiocin	0(0.0)	1(5.9)	3(33.3)	0(0.0)	1(1.0)	2(2.6)	7(2.4)
Gentamicin	1(6.7)	1(5.9)	0(0.0)	24(32.9)	49(47.6)	31(41.3)	106(36.3)
Rifampicin	0(0.0)	1(5.9)	0(0.0)	10(13.7)	19(18.4)	5(6.7)	35(12.0)
Mupirocin	1 (6.7)	0(0.0)	0(0.0)	9(12.3)	8(7.8)	7(9.3)	25(8.6)
Linezolid	0(0.0)	0(0.0)	0(0.0)	0(0.0)	1(1.0)	0(0.0)	1(0.3)
Vancomycin	0(0.0)	0(0.0)	0(0.0)	0(0.0)	0(0.0)	0(0.0)	0(0.0)

SE: *Staphylocuccus epidermidis*; SH: *Staphylocuccus haemolyticus*; OT: Other staphylococcal species. Species distribution in other species were *S*. *capitis*, *S*. *warneri*, *S*. *cohnii*, *S*. *pasteuri*, *S*. *caprae*, *S*. *hominis*, *S*. *saprophyticus*, *S*. *nepalensis* and *Staphylococcus* spp.

**Table 3:** Presence of biofilm production and adhesion genes in MR-CoNS.

Biofilm formation	Community environments			Total (%) n = 41	Hospital environments			Total (%) n = 251
	SE (%) n = 15	SH (%) n = 17	OT(%) n = 9		SE (%) n = 73	SH (%) n = 103	OT (%) n = 75	
**CRA**								
Red (%)	0(0.0)	1(5.9)	3(33.3)	4(9.8)	4(5.5)	2(1.9)	17(22.7)	23(9.2)
Black (%)	3(20.0)	10(58.8)	2(22.2)	15(36.6)	35(47.9)	85(82.5)	28(37.3)	148(59.0)
Very black (%)	12(80.0)	6(35.3)	4(44.4)	22(53.7)	34(56.6)	16(15.5)	30(40.0)	80(31.9)
**MTP**								
Negative (%)	5(33.3)	4(23.5)	4(44.4)	13(31.7)	2(2.7)	15(14.6)	2(2.7)	19(7.6)
Low grade positive (%)	10(66.7)	8(47.1)	5(55.6)	23(56.1)	46(63.0)	64(62.1)	56(74.7)	166(66.1)
Highly positive (%)	0(0.0)	5(29.4)	0(0.0)	5(12.2)	25(34.2)	24(23.3)	17(22.7)	66(26.3)
**Adhesion genes**								
*ica*AD (%)	0(0.0)	0(0.0)	0(0.0)	0(0.0)	28(38.4)	7(6.8)	39(52.0)	74(29.5)
*bap* (%)	0(0.0)	0(0.0)	0(0.0)	0(0.0)	0(0.0)	3(2.9)	11(14.7)	14(5.6)
*fnb*A (%)	13(86.7)	4(23.5)	4(44.4)	21(51.2)	49(67.1)	41(39.8)	24(32.0)	114(45.4)
*mec*A (%)	15(100)	17(100)	9(100)	41(100)	73(100)	103(100)	75(100)	251(100)

SE: *Staphylocuccus epidermidis*; SH: *Staphylocuccus haemolyticus*; OT: Other staphylococcal species. Species distribution in other species were *S*. *capitis*, *S*. *warneri*, *S*. *cohnii*, *S*. *pasteuri*, *S*. *caprae*, *S*. *hominis*, *S*. *saprophyticus*, *S*. *nepalensis* and *Staphylococcus* spp.

### Determination of biofilm production by MR-CoNS

**Fig 1:**
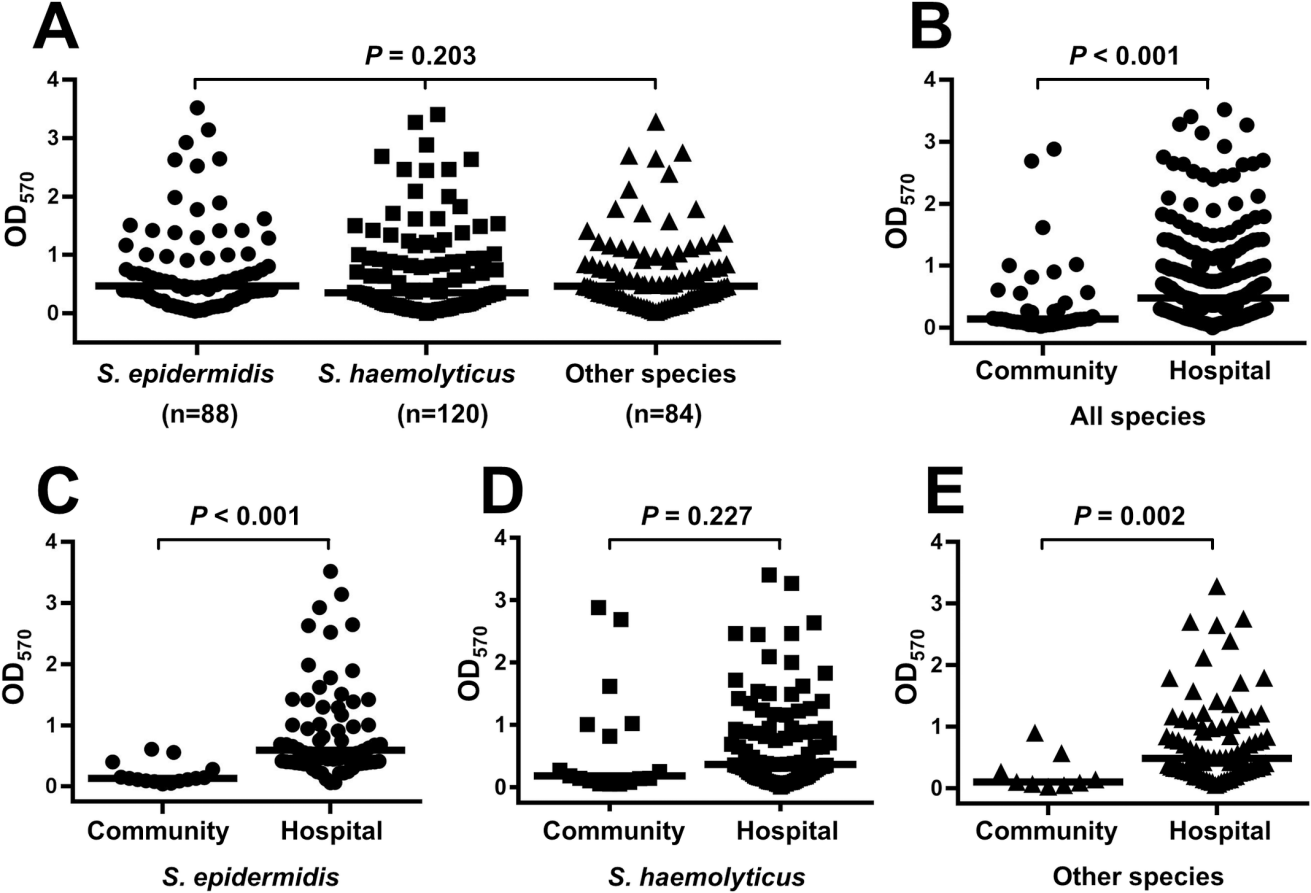
Biofilm producing ability of MR-CoNS obtained from community and hospital environments. (A) (B) (C) (D) and (E) Two group comparisons of median OD_570_ were analyzed by using Mann–Whitney U-tests (*P*-values < 0.05 indicate the statistical differences). (A) The comparison of OD_570_ among *S*. *epidermidis*, *S*. *haemolyticus* and other staphylococcal species. (B) (C) (D) and (E) the comparison of OD_570_ of MR-CoNS species between community and hospital isolates. Species distribution in other species were *S*. *capitis*, *S*. *warneri*, *S*. *cohnii*, *S*. *pasteuri*, *S*. *caprae*, *S*. *hominis*, *S*. *saprophyticus*, *S*. *nepalensis* and *Staphylococcus* spp.

The result of biofilm production of MR-CoNS by CRA method and MTP assay are demonstrated in Table 3 and Fig 1. Using the CRA method, 4 (9.8%) of 41 MR-CoNS isolates from community environments formed red colonies, 15 (36.6%) formed black colonies, and 22 (53.7%) formed very black colonies. Among 251 MR-CoNS isolated from hospital environments, 23 (9.2%) formed red colonies, 148 (59.0%) formed back colonies and 80 (31.9%) formed very back colonies. The biofilm production, tested by MTP, showed that 28 of the 41 MR-CoNS isolated from community environments were biofilm producers—including 23 (56.1%) isolates which had low grade positive and 5 (12.2%) isolates which were highly positive. Among 251 of MR-CoNS isolates from hospital environments, 232 were determined as biofilm producers, which categorized as low grade positive (66.1%) and high grade positive (26.3%).

### Detection of the *ica*AD, *bap* and *fnb*A genes

The *ica*AD gene was detected in 29.5% (74/251) of MR-CoNS isolates from hospital environments, classified into 38.4% (28/73) of *S*. *epidermidis*, 6.8% (7/103) of *S*. *haemolyticus*, and 52.0% (39/75) of other species (Table 3). The *bap* gene was found in 5.6% (14/251) of MR-CoNS isolated from hospital environments contained *S*. *haemolyticus* (2.9%) and other staphylococci species (14.7%). However, *bap* gene and *ica*AD genes were not detected in MR-CoNS isolated from community environments, while 51.2% (21/41) of MR-CoNS isolated from community and 45.4% (114/251) of MR-CoNS isolates from hospital environments were found to carry *fnb*A gene (Table 3). Overall, the prevalence of *fnb*A gene presented in *S*. *epidermidis*, *S*. *haemolyticus*, and other staphylococcal species was 70.5% (62/88), 37.5% (45/120) and 33.3% (28/84), respectively. The prevalence of *fnb*A gene in each specie distributed in different regions is shown in Table 3.

### Association of biofilm phenotypes, slime production and virulence genes in MR-CoNS obtained from difference sources

The comparison of biofilm biomass (median OD_570_) of MR-CoNS isolated from different areas revealed that strains obtained from hospital environments significantly produced more biofilm than those isolated from community environments (0.48 (0.28, 1.00) versus 0.14 (0.09, 0.40), respectively; *P*<0.001) (Fig 1B). We also compared the biofilm forming ability of each species separately as shown in Fig 1C–1E. The biofilm biomass of hospital-derived isolates of *S*. *epidermidis* was higher than that of community environmental isolates (0.61 (0.40, 1.17) versus 0.13 (0.09, 0.28), respectively; *P*<0.001). Likewise, other staphylococcal species of hospital environments had greater capacity to form biofilms (0.48 (0.29, 0.970)) compared with those of community environmental isolates (0.10 (0.07, 0.26), Fig 1E). In contrast, no statistical difference in biofilm production was observed between the hospital and community environmental isolates of *S*. *haemolyticus* (0.37 (0.16, 0.95) versus 0.18 (0.10, 1.01), respectively; *P*>0.05).

**Fig 2:**
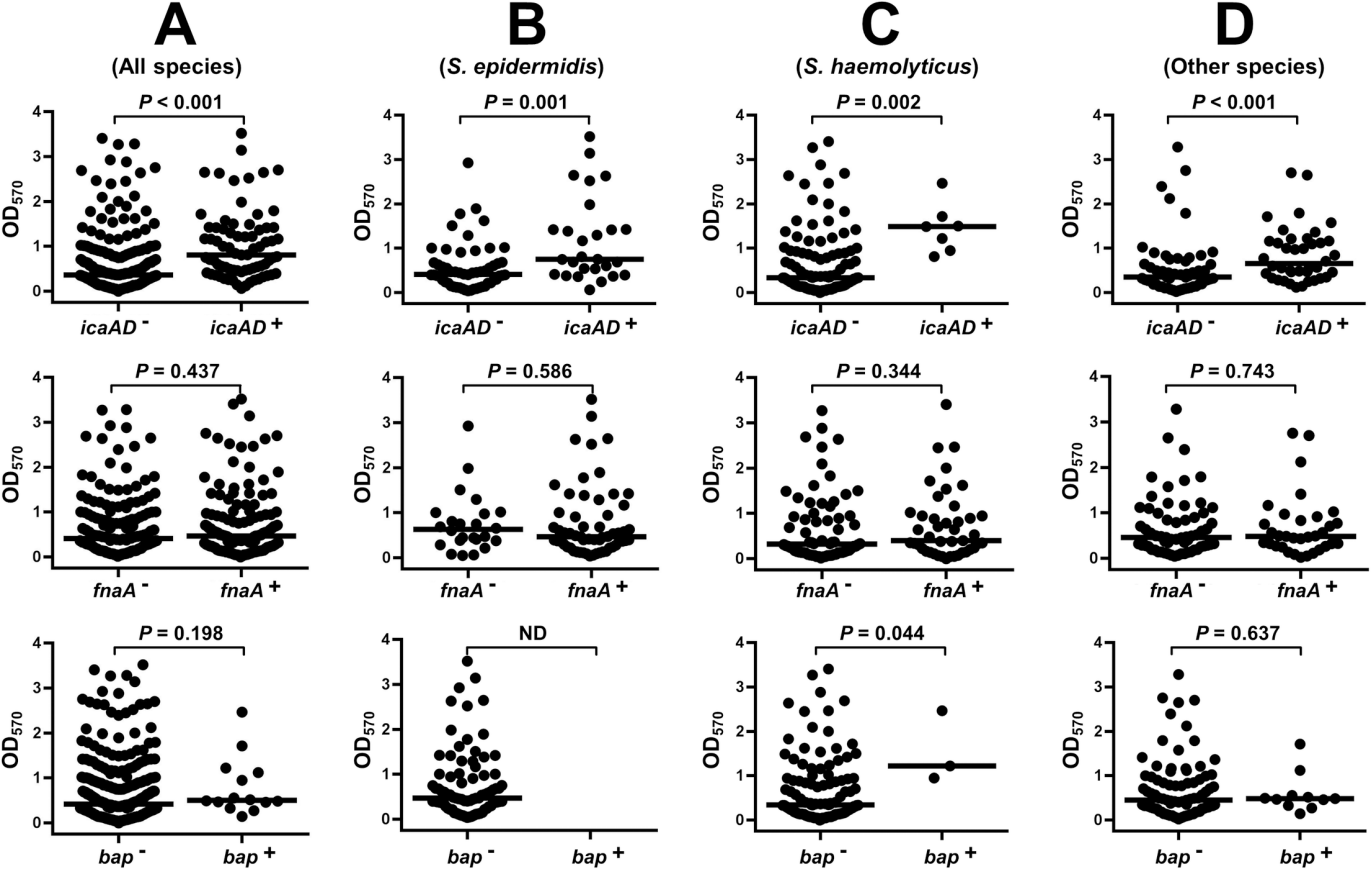
Comparisons of biofilm forming ability between the present and absent of each virulence genes. (A) (B) (C) (D) *P*- values represent the comparisons of median OD_570_ between two groups of MR-CoNS (Mann–Whitney U-tests, *P* < 0.0). Species distribution in other species were *S*. *capitis*, *S*. *warneri*, *S*. *cohnii*, *S*. *pasteuri*, *S*. *caprae*, *S*. *hominis*, *S*. *saprophyticus*, *S*. *nepalensis* and *Staphylococcus* spp.

The correlation between the present of each virulence gene and the biofilm phenotype of MR-CoNS isolates was statistically evaluated. Among the tested virulence genes, only *ica*AD were associated with biofilm formation on a plastic surface (Fig 2A–2D). MR-CoNS isolates harboring *ica*AD exhibited a significant increase in biofilm formation compared with those that lacked *ica*AD (0.81 (0.46, 1.41) versus 0.37 (0.15, 0.79), respectively; *P*<0.001) (Fig 2A). The data of biofilms and virulence genes were analyzed separately for each species group. We also found that *ica*AD-positive isolates of *S*. *epidermidis*, *S*. *haemolyticus*, and other species significantly made more biomass than strains without this gene (Fig 2B–2D). In contrast to *ica*AD, the presence of *fnb*A or *bap* (except *bap* of *S*. *haemolyticus*) genes were not statistically associated with biofilms producer phenotype (Fig 2A–2D). The *S*. *haemolyticus* carrying *bap* produced stronger biofilms than those negative for this gene (*P* < 0.05; Fig 2C).

The slime production on the CRA of MR-CoNS isolates was divided into three patterns: red, black, and very black and this contributed to the trend of increased biofilm production (Table 4). The median OD_570_ of isolates formed very black colonies and was of the highest value among three patterns with *P*-value of less than 0.05 (Table 4). When comparing the results of both the CRA and the MTP methods, the correlation of both methods was found.

**Table 4:** The ability to produce slime and biofilms (OD_570_) of MR-CoNS isolates.

Patterns of slime production	No. of isolates (%)	Median (IQR) of OD_570_	*P*-value ^*a*^
Red	27 (9.25)	0.328 (0.215, 0.516)	0.0156
Black	163 (55.82)	0.402 (0.181, 0.916)	
Very black	102 (34.93)	0.549 (0.285, 1.212)*	

^a^ *P*- value represents the comparisons of median OD_570_ among three slime production patterns of MR-CoNS (Kruskall Wallis test, *P* < 0.05). The Arthritis (*) indicates significant differences of biofilm forming ability between very black and red groups (Dunn's test).

## Discussion

The coagulase negative staphylococci (CoNS) are the normal flora of the human body. The main aim of the current study was to determine the biofilm production of MR-CoNS isolates from community and hospital environments. The prevalence of MR-CoNS isolated from community and hospital environments was 20.5% and 70.1%, respectively. The prevalence in hospital environment was very high compared to a previous study reported by Shobha, Rao, & Thomas (2005) [20]. The prevalence of staphylococci isolated from community was higher than that isolated from outdoor environments in Istanbul, Turkey [21], and public restrooms in London UK [22], but not different when compared to the prevalence isolated from hotels [23]. However, it was lower than the prevalence isolated from computer keyboards [24]. All MR-CoNS belonged to *S*. *haemolyticus* (41.1%), *S*. *epidermidis* (30.1%), and other staphylococcal species (28.8%). The prevalence of *S*. *haemolyticus* was high in the environment, with the tendency to develop the resistance to multiple antibiotics [25], while *S*. *epidermidis* was the predominant bacteria among CoNS isolated from patients because of its ability to form the biofilm on different surfaces [26].

High prevalence rate of multidrug-resistant staphylococci was found in this study. All MR-CoNS isolates harbored *mec*A gene. However, we found 15 *mec*A positive isolates that were not resistant to cefoxitin. This may be explained as not all *mec*A positive staphylococci are resistant to penicillin or cefoxitin due to the low expression of PBP2a protein causing the low levels of MIC as described previously [23].

Biofilm producing staphylococci are difficult to treat clinically because of the decrease of antibiotic sensitivity and host immune response [27]. We found that 90.8% of MR-CoNS isolated in this study were biofilm producer tested by the CRA method. This result was higher than the findings reported by Martini et al **(2016)**, which found that 43.75% biofilm producer of all CoNS isolated form platelet concentrates bags [28]. Oliveira and Cunha reported that 75% of clinical staphylococci isolates were biofilm positive determined by CRA method [29]. By using the MTP method to determine the biofilm production, we found that 24.3% of the isolates were highly positive, and 64.7% were low grade positive. This prevalence was higher than the biofilm producer staphylococci isolated from food, food environments [30], blood culture [31], various clinical specimens, and nasal samples [32]. Using the MTP method, the biofilm production of *S*. *epidermidis* and other staphylococci species obtained from hospital environments was found to be significantly higher than community environments. This result was supported by two previous studies. Wojtyczka et al **(201**4) [12] revealed that 37.5% of *S*. *epidermidis* isolated from hospital environments produced biofilm, while only 6.3% of *S*. *epidermidis* isolated from healthy people in Shanghai area of China was found to be biofilm producers [13]. We found no difference of biofilm production between *S*. *haemolyticus* isolated from hospital and from community environments. To our knowledge, this is the first comparison that found no significant different between *S*. *haemolyticus* isolated hospital environments and community environments.

We used both phenotypic and genotypic methods to determine the biofilm production. The *ica*AD and *bap* genes were detected in 29.5% (74/251) and 5.6% (14/251) of MR-CoNS isolated from hospital environments. On the other hand, the MR-CoNS isolates from community environments did not possess *ica*AD and *bap* genes. We also found that *ica*AD positive isolates of *S*. *epidermidis*, *S*. *haemolyticus*, and other staphylococcal species were significantly associated with the biofilm biomass. This is because *ica*AD gene encoded PIA or PNAG has been reported to play a significant role in biofilm formation in staphylococci [33]. Similar to this result, 81% of the biofilm producer staphylococci isolated from patients and healthy people were tested to carry the *ica*AD gene [29]. Previous studies reported that the *ica*AD gene was not detected in all biofilm producer of MR-CoNS isolates [34, 35], and this correlated with our finding that the *ica*AD gene has not found in biofilm producer of MR-CoNS isolated from community environments. It suggested that these biofilm producer strains used other systems, such as teichoic acids, to form biofilm [36].

Additionally, this study showed that the *bap* gene is present in MR-CoNS from different sources, but also showed that the presence of *bap* gene did not always correlate with biofilm production in MR-CoNS isolates. Similar to the study of Płoneczka-Janeczko et al. (2014) that the *bap* gene was not detected in 96.2% (51/53) biofilm producing *S*. *epidermidis* isolates from feline conjunctiva [37]. Although *bap* gene have been involved in biofilm formation, their presence is not absolutely necessary to the biofilm process. Additionally, the adhesion genes (*ica*AD and *bap*) of all MR-CoNS isolated in this study were found to be different between the community and the hospital environments, and the phenotypic traits of biofilm production were identical.

In conclusion, the present study found the high prevalence of staphylococci producing biofilm, particularly on hospital environments such as medical trolleys, intravenous poles, patient beds, wash basins, door handles, stethoscopes, nurse stations and laboratory clothes. The hospital isolates biofilm biomass was higher than community environment isolates. We also found that *ica*AD gene was associated with the biofilm formation tested by MTP, while *bap* was not determined to have the association. These results indicated the persisting ability of MR-CoNS in both hospital and community environments.

## Supporting information

S1 Table Specie distribution of MR-CoNS isolated from hospital and community environments. (DOCX)
