# Supplementary material for: Biofilm formation of methicillin-resistant coagulase negative staphylococci (MR-CoNS) isolated from community and hospital environments
Source: PLoS One. 2017 Aug 31;12(8):e0184172. doi: 10.1371/journal.pone.0184172 (PMC5578677; doi:10.1371/journal.pone.0184172)
Supplement: S1 Table — (DOCX) [file pone.0184172.s001.docx]

| **Species** | **N**  **n = 292** | **Community**  **n = 41** | **Hospital**  **n = 251** |
| --- | --- | --- | --- |
| *S. haemolyticus* | 120(41.1%) | 17(41.5%) | 103(41.0%) |
| *S. epidermidis* | 88(30.1%) | 15(36.6%) | 73(29.1%) |
| *S. capitis* | 33(11.3%) | 0(0.0%) | 33(13.1%) |
| *S. warneri* | 28(9.6%) | 5(12.2%) | 23(9.2%) |
| *S. cohnii* | 8(2.7%) | 2(4.9%) | 6(2.4%) |
| *S. pasteuri* | 3(1.0%) | 0(0.0%) | 3(1.2%) |
| *S. caprae* | 2(0.7%) | 0(0.0%) | 2(4.9%) |
| *S. hominis* | 2(0.7%) | 1(2.4%) | 1(0.4%) |
| *S. saprophyticus* | 2(0.7%) | 1(2.4%) | 1(0.4%) |
| *S. nepalensis* | 1(0.3%) | 0(0.0%) | 1(0.4%) |
| *Staphylococcus* spp*.* | 5(1.7%) | 0(0.0%) | 4(1.6%) |

**Supplemented data 1**: Specie distribution of MR-CoNS isolated from hospital and community environments
